# Commentary: Bibliometric analysis of targeted immunotherapy for osteosarcoma-current knowledge, hotspots and future perspectives

**DOI:** 10.3389/fimmu.2025.1590342

**Published:** 2025-07-16

**Authors:** Nan Zhang, Wanqing Li, Haiyang Wu, Qiang Guo

**Affiliations:** ^1^ Department of Tumor Ward, The Third Affiliated Hospital of Zhengzhou University, Zhengzhou, China; ^2^ Department of Operating Room, Xiangyang Central Hospital, Affiliated Hospital of Hubei University of Arts and Science, Xiangyang, China; ^3^ Department of Orthopaedics, The First Affiliated Hospital of Zhengzhou University, Zhengzhou, China; ^4^ Department of Orthopaedics, Baodi Hospital Affiliated to Tianjin Medical University, Tianjin, China

**Keywords:** osteosarcoma, immune function, immunotherapy, immune checkpoint inhibitor, targeted therapy

Bibliometrics, a sub-discipline of library and information science, employs mathematical and statistical methods to quantify various characteristics of documents, thereby describing, evaluating, and forecasting the current state and future trends of scientific and technological progress (1, 2). In recent years, the exponential growth in biomedical literature has garnered significant attention for bibliometrics as a method capable of quantitatively and qualitatively analyzing research trends and hotspots within a given discipline. Take osteosarcoma as an example, our preliminary statistics indicate that there have already published over 14 bibliometric articles related to osteosarcoma (3–7). However, it is noteworthy that despite the increasing number of these articles, there is substantial variability in their research methodologies, impacting the comparability and rigor of the results (3–7).

Furthermore, unlike meta-analyses and other clinical studies that are guided by established protocols or checklists, bibliometrics lacks a definitive international guideline (8, 9). This absence creates challenges for researchers in selecting methodologies, processing data, and interpreting results. In this context, we aim to examine the current issues in bibliometrics, using a recently accepted article by the *Frontiers in Immunology* as a case study. We also call for multidisciplinary collaboration to develop a universally accepted bibliometric guideline, enhancing the consistency and reliability of research.

We read with great interest on the publication by Hu et al. (10) titled “Bibliometric analysis of targeted immunotherapy for osteosarcoma-current knowledge, hotspots and future perspectives”, which has published in the issue of *Frontiers in Immunology*. By using bibliometrics, it highlights the global trends in the application of targeted immunotherapy in the field of osteosarcoma, and offers insights into current challenges and future directions in this area. Overall, this study reported the annual publications, main contributors including countries/regions, institutions and authors, core journals and references, as well as popular keywords and their change trend in this domain. As the authors state, tumor immunotherapy research has become a high-profile area of osteosarcoma, the importance of this study deserves to be recognized. However, in terms of the paper retrieval and software analysis process, we would like to discuss with authors as follows:

Firstly, we agree with the authors’ use of Web of Science Core Collection (WOSCC) database for data retrieval. Most previous studies have considered WOSCC to be one of the most suitable databases for bibliometric analysis (11, 12). However, to the best of our knowledge, WOSCC is a comprehensive citation indexing database comprising multiple sub-databases including SCI-EXPANDED, SSCI, Arts & Humanities Citation Index (AHCI), Conference Proceedings Citation Index - Science (CPCI-S), Conference Proceedings Citation Index - Social Science & Humanities (CPCI-SSH), and so on. In our opinion, the inclusion of all these sub-databases for sourcing eligible articles might not align optimally. Of them, SCI-EXPANDED emerges as the potential optimal choice for conducting bibliometric analyses. And in most previous bibliometric studies, many authors chose the data form SCI-EXPANDED as their data source. Consistent with this view, several scholars also believe that it is inappropriate to combine the use of these different types of databases in one bibliometric analysis (13, 14). However, in this study, the authors did not further explain the use of the WOSCC sub-databases. If all sub-databases were used, the necessity worth further discussion.

Secondly, in this study, the author use “TS” as the field tags. According to WOSCC, TS refers to topic search that comprises the title (TI), abstract (AB), author keywords (AK), and keyword plus (KP) terms. As for keywords, AK means keywords are provided by the authors, while KP are those automatically extracted by the system. In our experience, KP might not be appropriate to include in the search process. Although TS could expand the scope of literature search, many unrelated literatures in this field will also be included. For example, when we use the search terms of Hu’s study (10) and the field tags including TI, AK, and AB to search for literature, only 465 relevant articles were obtained from 2000 to 2023. Moreover, after we checked the literature abstracts and contents in Table 1 of Hu’s study (10), which has summarized the top 10 core literatures in the field of targeted immunotherapy for osteosarcoma, at least five (50%) of the articles do not correspond to the research topic (15–19). Such a high proportion of unrelated literature not only distorts citation analysis and co-citation networks but also undermines the validity of subsequent bibliometric mapping and clustering analyses. The inclusion of irrelevant articles can artificially inflate certain research areas, create false thematic connections, and ultimately lead to misleading conclusions about the intellectual structure and research trends in the field. Therefore, while TS searches may appear comprehensive, their tendency to capture unrelated literature poses a significant threat to the accuracy and reliability of bibliometric studies.

In addition, search terms are also very important because some medical terms represent the same thing although they have different forms. In this study, the author only used the term of “immunotherapy” to find immunotherapy-related datasets. We believe this term could not totally identified all related studies and many potentially relevant papers may be missed. In our opinion, the author also should add the following terms into the search formula including “immune checkpoint blockade*”, “immune checkpoint inhibitors *”, “CTLA-4”, “PD-L1”, “PD-1” and so on. Moreover, as many terms have plural and singular alternation. The author could use several wildcards such as “*”. The wildcard “*” means it could in place of any number of characters. For example, “immunotherap*” would also return the terms of “immunotherapy” and “immunotherapies” (**Table 1**).

**Table 1:** Recommended Protocols for Bibliometric Analysis in Medical Research.

Category	Best practice	Rationale
Database Selection	Use SCI-EXPANDED instead of full WOSCC for medical topics.	Avoids noise from non-SCI sub-databases (e.g., AHCI, CPCI-SSH) that may distort citation metrics
Search Strategy	Avoid Topic Search (TS) for broad terms; refine using Title (TI), Abstract (AB) and Author Keywords (AK).	TS often captures off-topic papers due to Keyword Plus (KP) mismatches.
Keyword Optimization	Include term variants (singular/plural, synonyms) and wildcards (*).	Ensures comprehensive coverage of the field (e.g., “immunotherap*” captures “immunotherapy”/”immunotherapies”).
Software Parameters	Display the specific parameter settings of the software	Ensure reproducibility of results

Last but not least, according to our knowledge, HisCite, CiteSpace and VOSviewer are all the most popular tools used to visualize scientific literature. The parameter settings significantly impact their outcomes. Choosing specific words or terms and assigning weights directly influences the study’s findings. Optimal keyword selection better captures the literature’s themes, directly impacting result accuracy and interpretability. Thus, it is crucial to sensibly select and adjust parameters when using HisCite, CiteSpace and VOSviewer for bibliometric analysis, considering research objectives and data characteristics. However, the authors did not explain the detailed parameter settings for HisCite, CiteSpace and VOSviewer in this study, hindering subsequent researchers from validating these outcomes.

First, node selection criteria constitute a primary factor influencing the visualization. For instance, in CiteSpace, the selection of node types (authors, institutions, keywords, cited references) and the threshold for the number of nodes per time slice (Top N or Top N%) directly alter the elements included in the network. Excessively high thresholds may omit emerging or potentially significant research entities, whereas overly low thresholds may introduce excessive noise, resulting in overly complex maps that obscure core structures. Similarly, in VOSviewer, the minimum occurrence threshold for entities such as keywords or authors (minimum number of occurrences of a keyword/author) serves to filter core elements while excluding low-frequency noise. Second, the configuration of time slicing and time span is critical for dynamic evolution analysis. A distinctive feature of CiteSpace lies in its time-zone and timeline views, which reveal the trajectory of knowledge development. The duration of time slices (years per slice) affects the sensitivity of burst detection and the stability of cluster labels. Excessively short time slices may fragment evolutionary trends, whereas overly long slices may obscure short-term significant changes. Many studies employing these tools mention the software versions and basic data sources but often lack detailed justification for specific parameter settings, such as the rationale for threshold selection or pruning methods. Such omissions not only compromise the reproducibility of findings but also hinder readers’ ability to evaluate the robustness of the analytical results.

Given the current landscape, we posit that the formulation of an international bibliometric guideline is of paramount importance. Such a guideline should encompass methodologies for the acquisition and processing of bibliometric data, definitions and applications of standard bibliometric indicators, and protocols for data analysis and interpretation. Moreover, the development of this guideline necessitates the collaboration and engagement of researchers from diverse disciplines, including library and information science, statistics, computer science, and medicine. These experts must collectively deliberate and address the pivotal issues inherent in bibliometric research. To advance this objective, we propose initiating discussions and calls for action on the formulation of bibliometric guidelines at international academic conferences and in scholarly journals. This initiative should invite the participation and insights of experts and scholars from pertinent fields. Furthermore, the establishment of an *International Bibliometrics Research Consortium* could significantly enhance collaboration and communication among researchers worldwide. This consortium would facilitate the sharing of research experiences and methodologies, thereby fostering the advancement of bibliometric science.

In summary, we congratulate the authors and colleagues for their exceptional comprehensive bibliometric study, providing a detailed analysis of research trends and key contributors in the field of targeted immunotherapy for osteosarcoma. Nevertheless, we believe that our suggestions regarding the search process and software parameter settings could assist the authors in obtaining more reliable analytical outcomes, thereby avoiding biases that might misguide scholars in this field. Additionally, bibliometric analysis has become an increasingly important and recognized methodology; however, there is still a lack of guidelines, akin to reviews or meta-analyses, to guide scholars in conducting proper data retrieval, analysis, and interpretation. Therefore, we advocate for increased collaboration among scholars to develop guidelines or expert consensus for the use of bibliometric analysis at the earliest opportunity.
